# Acknowledgement to Reviewers of *Proteomes* in 2013

**DOI:** 10.3390/proteomes2010084

**Published:** 2014-02-27

**Authors:** Proteomes Editorial Office

**Affiliations:** MDPI AG, Klybeckstrasse 64, CH-4057 Basel, Switzerland

The editors of *Proteomes* would like to express their sincere gratitude to the following reviewers for assessing manuscripts in 2013:
Addis, Maria FilippaGuzman, NorbertoQuintana, Luís F.Alterman, MichailHardt, MarkusQuong, Andrew A.Aman, PierreHasnain, GhulamRakus, DariuszArmengaud, JeanHassanein, MohamedRepetto, OmbrettaAzmi, Asfar S.Huang, Sheng-YuRivoltini, LiciaBenedetti, Celso EduardoHwang, Sun-IlRuhl, StefanBlanch, Ewan W.Jeffrey, WadeSalzet, MichelBrown, Celeste JJones, AlexSchanstra, Joost P.Burg, DominicKim, Sun TaeSchiller, Martin R.Carpentier, SebastienKobel, MartinSchilling, OliverChambery, A.Lalowski, MaciejSkvortsova, IraChang, Chih-chingMagni, FulvioSussman, MikeCillero-Pastor, BertaMahmood, TariqSze, NewmanCunha, AngelaMathivanan, SureshTaylor, Nicolas L.Dawson, Kenneth A.Metzger, JochenTucker, RichardFarrah, TerryMorgan, William F.Uversky, VladimirFleming, Jodie MMundy, JohnUwe, ChristiansFriedewald, JohnNylund, ReettaVitorino, RuiGomez, DavidOstroff, Rachel M.Webb, Lauren J.Gosti, FrançoisePereira, Ricardo N.Wiig, HelgeGromov, PavelPesta, DominikWilmarth, Phillip


